# The Microbiome of Brazilian Mangrove Sediments as Revealed by Metagenomics

**DOI:** 10.1371/journal.pone.0038600

**Published:** 2012-06-21

**Authors:** Fernando Dini Andreote, Diego Javier Jiménez, Diego Chaves, Armando Cavalcante Franco Dias, Danice Mazzer Luvizotto, Francisco Dini-Andreote, Cristiane Cipola Fasanella, Maryeimy Varon Lopez, Sandra Baena, Rodrigo Gouvêa Taketani, Itamar Soares de Melo

**Affiliations:** 1 Department of Soil Science, “Luiz de Queiroz” College of Agriculture, University of São Paulo, Piracicaba, São Paulo, Brazil; 2 Department of Biology, Pontificia Universidad Javeriana, Bogotá, Distrito Capital, Colombia; 3 Center of Biotechnology and Bioindustry, Colombian Corporation of Agricultural Research (CORPOICA), Bogotá, Distrito Capital, Colombia; 4 Colombian Center for Genomic and Bioinformatics from Extreme Environments (GeBiX), Bogotá, Distrito Capital, Colombia; 5 Laboratory of Environmental Microbiology, EMBRAPA Environment, Jaguariúna, São Paulo, Brazil; U. S. Salinity Lab, United States of America

## Abstract

Here we embark in a deep metagenomic survey that revealed the taxonomic and potential metabolic pathways aspects of mangrove sediment microbiology. The extraction of DNA from sediment samples and the direct application of pyrosequencing resulted in approximately 215 Mb of data from four distinct mangrove areas (BrMgv01 to 04) in Brazil. The taxonomic approaches applied revealed the dominance of *Deltaproteobacteria* and *Gammaproteobacteria* in the samples. Paired statistical analysis showed higher proportions of specific taxonomic groups in each dataset. The metabolic reconstruction indicated the possible occurrence of processes modulated by the prevailing conditions found in mangrove sediments. In terms of carbon cycling, the sequences indicated the prevalence of genes involved in the metabolism of methane, formaldehyde, and carbon dioxide. With respect to the nitrogen cycle, evidence for sequences associated with dissimilatory reduction of nitrate, nitrogen immobilization, and denitrification was detected. Sequences related to the production of adenylsulfate, sulfite, and H_2_S were relevant to the sulphur cycle. These data indicate that the microbial core involved in methane, nitrogen, and sulphur metabolism consists mainly of *Burkholderiaceae*, *Planctomycetaceae, Rhodobacteraceae*, and *Desulfobacteraceae*. Comparison of our data to datasets from soil and sea samples resulted in the allotment of the mangrove sediments between those samples. The results of this study add valuable data about the composition of microbial communities in mangroves and also shed light on possible transformations promoted by microbial organisms in mangrove sediments.

## Introduction

Mangrove ecosystems constitute a large portion (60–70%) of the coastline in the tropical and subtropical regions of Earth. In the Americas, they cover approximately 4.1 million hectares [1] and are located at the interface between oceanic and continental waters [2]. The mangrove ecosystem is essential for maintenance of sea level and for protection of the coast [3]. Environmental conditions particular to this biome are the salinity, which is related to the proximity to the sea, and the frequent anaerobic condition caused by tidal variation [4], [5], which results in a redox potential that ranges from –200 to +150 mV [6]. Such conditions make mangroves hotspots for microbial diversity, and the microbial community plays essential roles in the functioning and maintenance of the ecosystem. For example, microbes engage in biogeochemical cycles and supply plants and animals with primary nutritional sources [7], [8]. Hence, microbial diversity and activity are fundamental for the productivity, conservation, and recovery of mangroves [9], [10].

The microbial community present in mangrove sediment is strongly influenced by biogeographical, anthropogenic, and ecological properties, including the food web in the ecosystem, nutrient cycling, and the presence of organic and inorganic compounds in the sediment [2]. In recent years, the microbial inhabitants of mangroves have been assessed using a range of techniques, including classical cultivation approaches, fingerprinting methods, and use of clone libraries to analyse phylogenetic and functional genes [9], [11]–[15]. Previous studies were conducted in sediments from pristine [13] or urban [16] mangroves, from areas affected or not affected by shrimp farms [17], and in mangrove systems contaminated by oil and industrial contaminants [18], [19]. More specifically, researches conducted by our group are based on mangrove sediments along the coastline of Sao Paulo State in Brazil, where several descriptions of the microbiology found in mangrove were made based on culture-dependent [12] and culture-independent approaches [13], [20]; see these references for descriptions of the chemical and physical features of the analysed mangroves. Briefly, these studies describe a very constant microbial community within each area assessed, and the shifting in the community content according to the state of preservation found in each mangrove targeted. However, to date, a comprehensive description of the microbial life in the mangrove ecosystem is lacking, and comparisons among distinct mangroves based on metagenomics might significantly contribute to a better overview of the functioning and resilience of mangroves.

Metagenomic analysis provides a method to evaluate the basis for potential metabolic pathways of this environment, representing a single snapshot, where the DNA present in the environment can be sequenced to provide the widest view of the microbial community in terms of both taxonomy and potential functioning [21], [22]. Such an approach provides a relatively unbiased view of the microbial diversity present in the system, and such data provide information about community structure and the genetic basis present in the environment [23]. In the last decade, metagenomic analyses supported by high throughput sequencing [24], [25] of environmental DNA have been widely used to detect microbial ecological properties [26], [27]. Metagenomic studies have been conducted in several ecosystems (as bioreactors, host-associated communities and natural environments), with a remark for those studies carried out on marine waters [28], [29], [30], [31], pristine and agricultural soils [32], [33], and extreme environments [34]–[37].

In this study we present a robust description of the microbes found in four different mangrove areas in São Paulo State, Brazil. These descriptions are based on metagenomic data obtained by direct 454-pyrosequencing of DNA collected from the mangrove sediment environment. This study describes the microbial groups present in these areas, the preferential metabolic processes that might be occurring in this ecosystem, and the biogeochemical cycles that are important for energy metabolism (i.e., carbon, nitrogen, and sulphur). The metagenome profiles of the Brazilian mangroves also are compared with profiles from other land and marine environments.

## Materials and Methods

### Ethics Statement

No specific permits were required for the described field studies. The studied locations are not privately-owned. Moreover, the study did not involve endangered or protected species. Indeed, the São Paulo Research Foundation (FAPESP) and Brazilian Agricultural Research Corporation (Embrapa) approved this study development.

### Sampled Mangroves and Composition of Datasets

**Figure 1:**
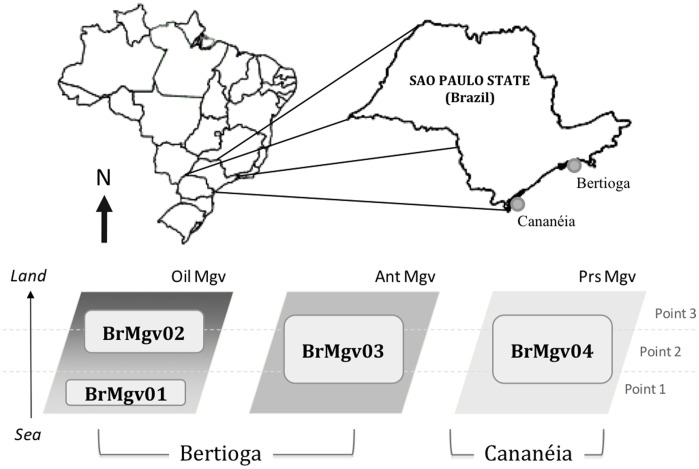
Location of mangroves and composition of each analysed dataset. Codes indicate the name attributed to the datasets analyzed by metagenome.

Three distinct mangroves (divided into four samples as described below; Table 1) located on the coast of São Paulo State, Brazil were the basis of this study. The first two mangroves are located close to the city of Bertioga (Figure 1). One of the mangroves from was affected by oil contamination (labelled Oil Mgv) due to an oil spill that occurred in 1983, when 35 million litres of oil were released into the mangrove area. This mangrove is easily divided in two subregions that are separated by a small stream that crosses the mangrove. In the area landward of the river (site BrMgv02), the oil effects are still present, even 28 years after the spill. In this mangrove, the native vegetation is still undergoing recovery. The area nearer to the sea in the same mangrove (site BrMgv01) does not show effects of the oil spill, possibly due to isolation from the oil drainage promoted by the stream. In this area the vegetation is more similar to that of the other mangrove located in the city of Bertioga. This other mangrove is located near the city centre, and it suffers the effects of sludge and other urban waste that enters the sea near the area (Ant Mgv; here called site BrMgv03). The third mangrove is located in the city of Cananéia (Prs Mgv), and it experiences the most pristine conditions found among the mangroves in this part of Brazil (site BrMgv04) (Table 1).

**Table 1:** Characteristics and history of contamination of the mangroves analysed in this study.

Mangrove metagenome	Description	City	Coordinates	Water	Contamination	Vegetation
BrMgv01	Area free of oil contamination in the spilled mangrove (Oil Mgv)	Bertioga	23°53′49′ S 46°12′28′ W	Mixture from seaand small rivers	Small impact ofoil spill	Presence of mangrove species*, predominance of *Rhizophora mangle*
BrMgv02	Area highly impacted by the oil contamination in the Oil Mgv	Bertioga	23°53′49′ S 46°12′28′ W	Mixture from seaand small rivers	Highly affectedby oil spill	Under recovery, low density of *R. mangle*
BrMgv03	Mangrove near the city, underanthropogenic pressure(Ant Mgv)	Bertioga	23°54′06′ S 45°15′03′ W	Mixture from seaand small rivers	From humanactivity	Abundant, existence of other species besides those typically found in mangroves
BrMgv04	Located in a preservation area,under pristine conditions(Prs Mgv)	Cananéia	25°05′02′ S 47°57′42′ W	Open sea	Very low	Abundant, but exclusively composed by mangrove species

* in the state of Sao Paulo, the mangrove forest is composed mainly of three species: *Avicennia shaueriana*, *Laguncularia racemosa*, and *Rhizophora mangle*.

Concerning the other characteristics of mangroves, the physicochemical parameters were previously determined, and published in other articles of our group [13], [19]. Briefly, variations in pH were small (5.9 to 7.1), and major differences are observed in the pristine mangrove (BrMgv04), where it is found higher contents of total carbon, organic carbon, and total nitrogen when compared with other areas [19]. Moreover, higher values for salinity were observed in the same mangrove due to the direct flood from the open sea (Table 1). Concerning the contamination level of oil spill in the areas, we can state that, approximately 29 years after the spill, the presence of oil is visible, mainly in the undersurface layers (up to 30 to 50 cm depth).

### Sequencing of Environmental DNA from Mangrove Sediments

A minimum of 5 µg of environmental DNA is needed to initiate the pyrosequencing protocol and to avoid extraction biases [31]. From each of the four mangrove areas, six sediment samples were obtained separately using a sediment core (7 cm diameter and 30 cm depth). From each of these core sediment samples (total n = 24), aliquots of homogenised sediment of 0.3 g were subjected to DNA extraction using the Power Soil DNA Isolation kit (MoBio® Laboratories Inc., Carlsbad, CA, USA). After the extractions, DNA from all six samples from each mangrove area were pooled together (approximately 20 ng µl^–1^ of DNA from each extraction – from a total of 100 µl), and the DNA was concentrated in a speed vacuum centrifuge (3,000 rpm for 30 min) to a final volume of 10 µl. A NanoDrop (Thermo Scientific, Wilmington, DE, USA) spectrophotometer was used to obtain an accurate quantification of the extracted DNA and to measure other important parameters for DNA quality, such as the ratio of absorbance at 260/280 nm and 260/230 nm. We have pooled samples based on previous results of microbial fingerprinting [13], [20], which revealed the great repeatability of communities profile when several samples were analyzed within of each area targeted in our metagenomic survey.

Environmental DNA samples from the Brazilian mangrove sediments were subjected to pyrosequencing using 454 GS FLX Titanium technology at Roche Applied Sciences (Indianapolis, IN, USA). One 454 plate was used, and DNA from each of the four mangrove areas constituted one-quarter of the plate. These samples were run at Agilent 2100 Bioanalyzer, in order to guarantee the quality and quantity necessary for a successful sequencing approach. Obtained sequences were subjected for quality trimming using an in-house python script with the following parameters: MinSeqSize = 30 pb, cutoff-quality = 20 measure with slice-windows of = 20 pb. The clean sequences were uploaded to the metagenomic RAST (MG-RAST) server and made publicly accessible under the codes 4451033.3, 4451034.3, 4451035.3, and 4451036.3 for mangroves BrMgv01, BrMgv02, BrMgv03, and BrMgv04, respectively. In addition, the complete dataset, which includes all of the sequences, has received the code 4452857.3.

### Extraction and Analysis of 16 S rDNA Sequences (SSU rDNA Sequences)

**Figure 2:**
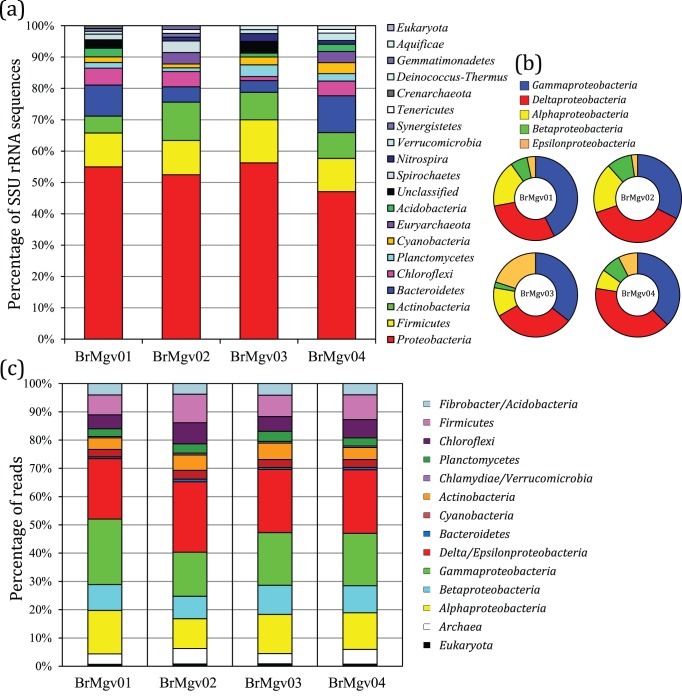
Taxonomic affiliation of metagenomic reads. (a) SSU rRNA sequences from the datasets were classified by BLASTN against the RDPII database using Classifier v 2.2 software. (b) Differential proportion of sequences assigned within the phylum *Proteobacteria*. (c) Results for complete datasets evaluated by BLASTX analysis against the SEED database using MG-RAST v 2.0 software. Others assignment methodologies are presented in the supplemental material (Supplementary Figure S1 and Table S1).

The SSU rDNA sequences were extracted from each dataset using a HMMER search against the Markov model based on multiple sequence alignment [38] and BLASTN [39] analysis against the RDPII database [40]. The ribosomal sequences retrieved were filtered, and those containing more than 50 bp were considered for taxonomic affiliation. The sequences were aligned using the NAST align tool at Greengenes database [41] (http://greengenes.lbl.gov/cgi-bin/nph-NAST_align.cgi) (batch size for NAST: 5; minimum percentage identity: 75). Subsequently, the sequences were classified taxonomically using the “classify a batch of sequences against multiple taxonomies” tool (http://greengenes.lbl.gov/cgi-bin/nph-classify.cgi). Classification of the sequences was performed using BLASTN (against nr/nt and with cuttoff E-value 1e-10) against the Greengenes, RDPII, and NCBI databases (Table 2) and also using Classifier v2.2 software (cut-off E-value of 1e-10) [42] with a confidence threshold of 80% against the RDPII database (Figure 2).

**Table 2:** Comparison of the taxonomic assignment of reads using different annotation systems.

		Number of classified reads (Proportion)				
Annotation system	Datasets	*Bacteria*	*Archaea*	*Eukaryota*	*Virus*	unclassified
**MG-RAST**	BrMgv01	77711 (32%)	2689 (1.1%)	506 (0.2%)	30 (0.01%)	169057 (67.6%)
**(BlastX vs. SEED)** a	BrMgv02	66044 (28%)	3390 (1.5%)	480 (0.2%)	39 (0.01%)	161280 (69.7%)
	BrMgv03	66733 (31%)	2292 (1.0%)	526 (0.2%)	49 (0.02%)	145321 (67.6%)
	BrMgv04	55438 (25%)	2730 (1.2%)	391 (0.2%)	16 (0.007%)	159030 (73.1%)
**WebCARMA**	BrMgv01	50180 (20%)	980 (0.4%)	1826 (0.7%)	0	197007(78.8%)
**(BlastX vs. Pfam)^ab^**	BrMgv02	47169 (20%)	1396 (0.6%)	1782 (0.7%)	0	180886 (78.2%)
	BrMgv03	109454 (51%)	1861 (0.9%)	4728 (2.2%)	0	98878 (46.0%)
	BrMgv04	103331 (47%)	3139 (1.4%)	3830 (1.8%)	0	107305 (49.3%)
**MEGAN 4.0**	BrMgv01	152642 (61%)	3071 (1.2%)	7377 (2.9%)	183 (0.07%)	86720 (34.7%)
**(Blastx vs. NCBInr)^ab^**	BrMgv02	125427 (54%)	3778 (1.6%)	6782 (2.9)	246 (0.1%)	95000 (41.0%)
	BrMgv03	117931 (55%)	2105 (1.0%)	6109 (2.8%)	219 (0.1%)	88577 (41.2%)
	BrMgv04	126742 (58%)	3986 (1.8%)	6453 (3.0%)	161 (0.07%)	80263 (36.8%)

a cut-off E-value 1e-10;

b LCA parameters (maximum number of match per read = 5, min support = 5, min score = 5, top percent = 10).

### Taxonomic Assignment of Metagenomic Sequences

The taxonomic assignment of unassembled clean metagenomic sequences was performed using BLASTX against the SEED and Pfam databases [39] on the MG-RAST server v2.0 (http://metagenomics.nmpdr.org) [43] and on the WebCARMA v1.0 online system (http://webcarma.cebitec.uni-bielefeld.de/cgi-bin/webcarma.cgi) [44], respectively, using a cut-off E-value of 1e-10. BLASTX was also used to conduct a similarity search against the NCBI-NR database, and MetaGenome Analyzer software (MEGAN v4.0) [45] with the LCA algorithm (maximum number of matches per read: 5, min support: 5, min score: 35, top percent: 10) was used to visualize results.

Statistical assessment of the data was performed using results from the MG-RAST v2.0 annotation system, and results were visualized using Statistical Analyses of Metagenomic Profiles (STAMP) software v 1.0 [46] in order to detect biologically relevant differences in the relative proportion of classified sequences. This analysis was performed using paired metagenomic samples (comparing one site to another individually) and statistical significance of the differences between samples was assessed by the Two-sided Fisher’s Exact test, and Storey’s FDR method was used for multiple test correction, as recommended by STAMP developers [46]. The most important taxa were selected by filtering by q-value (0.05), and using only those categories that had at least 100 sequences and more than 2-fold ratio between the proportions, as previously reported by Ghai et al. [47].

### Functional Analysis Using COG, KEGG, and SEED Identifiers

Functional classification was conducted using BLASTX (cut-off E-value of 1e-10) against COGs [48], which was downloaded from the NCBI ftp site and GenBank (nr/nt) local databases. BLASTX (cut-off E-value of 1e-5) and subsystem technology were used against the SEED-NR database in the MG-RAST v2.0 platform for functional sequence annotation. Annotation results for BLASTX against NCBI-NR were loaded into MEGAN v4.0, and classification was achieved using KEGGs [49] (http://www.genome.jp/kegg/) and SEED identifiers (Supplementary Table S2).

### Metabolic Mapping of the Methane, Nitrogen, and Sulphur Transformations in Mangroves

The main transformations of methane, nitrogen, and sulphur were analysed in the four mangrove datasets based on the KEGGs maps, where the number of sequences from each mangrove involved in each transformation was recorded. The resulting maps also indicate the abundance of each KEGGs step in the mangrove sediment metagenomes. This information was obtained using BLASTX against the NCBI-NR database and analysis using MEGAN v4.0.

Sequences assigned to methane, nitrogen, and sulphur transformations were extracted from datasets and affiliated with taxonomic groups to provide insights into the major microbial groups involved in the transformations of core compounds in mangrove sediments. Sequences associated with these nutrient transformations were affiliated with taxonomic groups by BLASTX at NCBI-NR database (cut-off E-value of 1e-5) and further taxonomic classification using MEGAN v4.0. The occurrence of distinct groups in the four mangrove metagenomes were visualized using a Venn diagram, and microorganisms involved in distinct cycles in all mangrove datasets were visualized using a similar clustering methodology.

### Comparison of Mangroves with Other Marine and Terrestrial Ecosystems

Taxonomic comparison among different datasets from various ecosystems (marine and terrestrial) (Supplementary Table S3) was performed using results generated by the MG-RAST v2.0 annotation system (cut-off E-value 1e -10). The results were first plotted to show the proportion of sequences assigned to specific taxa, and the frequency of taxonomic groups in each metagenome was used for the clustering analysis. The data were first submitted to a detrended correspondence analysis to check the distribution of data [50], then analysed using linear models (first gradient 2.131), and then subjected to principal component analysis performed using Canoco v4.52 [51].

## Results and Discussion

To our knowledge, this is the first description of the metabolic pathways found in microbes living in tropical mangrove sediments determined using pyrosequencing and metagenomics. We generated a metagenome dataset using the 454 technology for DNA sequencing that contains 905,521 sequences with an average length of 236 bases, which adds up to a total of 215.72 Mb. The total numbers of trimmed valid sequences obtained for each mangrove area were 249,993 for BrMgv01 (average size 235.2 bases, 55.75% GC content), 231,233 for BrMgv02 (average size 238.2 bases, 54.64% GC content), 214,921 for BrMgv03 (average size 247.9 bases, 56.36% GC content), and 217,605 for BrMgv04 (average size 222.9 bases, 54.66% GC content).

### Microbial Diversity in Mangroves Based on SSU rDNA Genes

A total of 358 partial sequences of SSU rDNA genes were found in the datasets, with values of 111, 82, 80, and 85 for BrMgv01, BrMgv02, BrMgv03, and BrMgv04, respectively. The numbers of sequences affiliated with each taxon were similar in each database (Supplementary Table S1), with a major abundance of *Proteobacteria* (47.1–56.3%), *Firmicutes* (10.5–13.8%), *Actinobacteria* (5.4–12.2%), *Bacteroidetes* (3.8–11.8%), and *Chloroflexi* (1.3–5.4%) (Figure 2a), followed by other minor groups represented by *Planctomycetes* (1.2–3.8%), *Cyanobacteria* (1.2–3.5%), *Acidobacteria* (0.0–2.7%), and Archaea (0–3.4%) (Figure 2a). Among the distinct mangrove sets, the following differences were observed: higher abundance of *Bacteroidetes* in BrMgv04, a lower number of sequences of *Chloroflexi* in BrMgv03, and higher occurrence of *Planctomycetes* in BrMgv03 (Figure 2a).

Focusing the present phylogeny analysis within the *Proteobacteria*, the numbers of sequences affiliated with distinct classes were similar among the four mangrove datasets (Figure 2b). The most frequent class detected was the *Gammaproteobacteria* (32.6–42.6%) (except for at BrMgv04), followed by *Deltaproteobacteria* (29.5–40.0%), *Alphaproteobacteria* (7.5–18.6%), *Betaproteobacteria* (2.2–9.3%), and *Epsilonproteobacteria* (2.3–20.0%) (Figure 2b). The dominance of the classes *Deltaproteobacteria* and *Gammaproteobacteria* corroborates the data reported by Dos Santos et al. [10], who also used pyrosequencing of 16 S rDNA tags and detected the dominance of these groups in mangroves under natural conditions and also after a simulated oil spill. The high occurrence of SSU sequences affiliated with *Deltaproteobacteria*, which are not commonly observed in metagenomes from seawater or soil samples, might be related to the mangrove ecosystem, where frequent anaerobic conditions could drive selection for specific microbial groups such as sulphate-reducing bacteria [14].

### Environmental DNA Affiliation with Distinct Databases

As an alternative to the taxonomic affiliations determined based on SSU rDNA sequences, phylogenetic analyses using the complete datasets were conducted by comparing the obtained sequences with sequences from different databases. From the total sequences obtained in this study, an average of 30.5% were classified using MG-RAST v2.0, 36.9% were classified using WebCARMA v1.0, and 61.5% were classified using MEGAN v4.0 based on GenBank BLAST analysis (Table 2). The higher rates of sequences affiliation in the last approach might be related to the higher number of available sequences in the reference database, and with the ability of LCA algorithm to affiliate sequences in high taxonomic levels, *e.g.* Bacteria domain. Contrastingly, other methodologies are more specific, using as the reference the available microbial genomes already published.

At the domain level, Bacteria were more abundant than Archaea in all four mangroves metagenome datasets. Within the total of 36.1% of sequences that matched the SEED database, 28.1% were considered to be Bacteria, and 1.2% and 0.2% were related to Archaea and Eukarya, respectively. The affiliations in the other databases were similar (Supplementary Figure S1), generating trends that are similar with most of available metagenomes, as for sea sediments [31] or soils [32]. However, it should be noted that particular environments (e.g., extreme environments) might harbour more cells affiliated with Eukarya or Archaea than Bacteria [35], [52].

A more detailed overview of the microbial groups present in mangrove sediments revealed the dominance of bacterial sequences affiliated with the *Delta*/*Epsilonproteobacteria* and *Gammaproteobacteria*, based on three systems used for taxonomic affiliation (BLASTX against the SEED and Pfam databases, and on the WebCARMA v1.0) (Figure 2c). Other less abundant groups were *Alphaproteobacteria* and *Bacteroidetes* (except for the affiliation based on the SEED database, which showed a lower number of *Bacteroidetes*-like sequences), followed by *Firmicutes*, *Actinobacteria*, and *Archaea* (mostly methanogenic *Euryarchaeota*). Minor groups were *Cyanobacteria*, *Chloroflexi*, *Fibrobacteres*/*Acidobacteria*, and *Eukarya* (Figure 2c). Comparison of the occurrence of the taxonomic groups between phylogenetic approaches (i.e., SSU rDNA affiliations and complete dataset assignment) revealed correlation values ranging from 0.96 to 0.98. The taxonomic groups found and their level of occurrence agreed with the data obtained by Gomes et al. [53], who assessed the diversity of bacteria in bulk sediments of mangroves in comparison with the rhizosphere. The major groups in their bulk samples were similar to those described herein, whereas the rhizosphere contained an increased percentage of *Acidobacteria*, *Actinobacteria*, *Verrucromicrobia*, *Burkholderiales*, *Caulobacterales*, and *Rhizobiales* and significantly lower relative abundances of *Chloroflexi*, *Firmicutes*, and *Desulfobacterales*.

**Figure 3:**
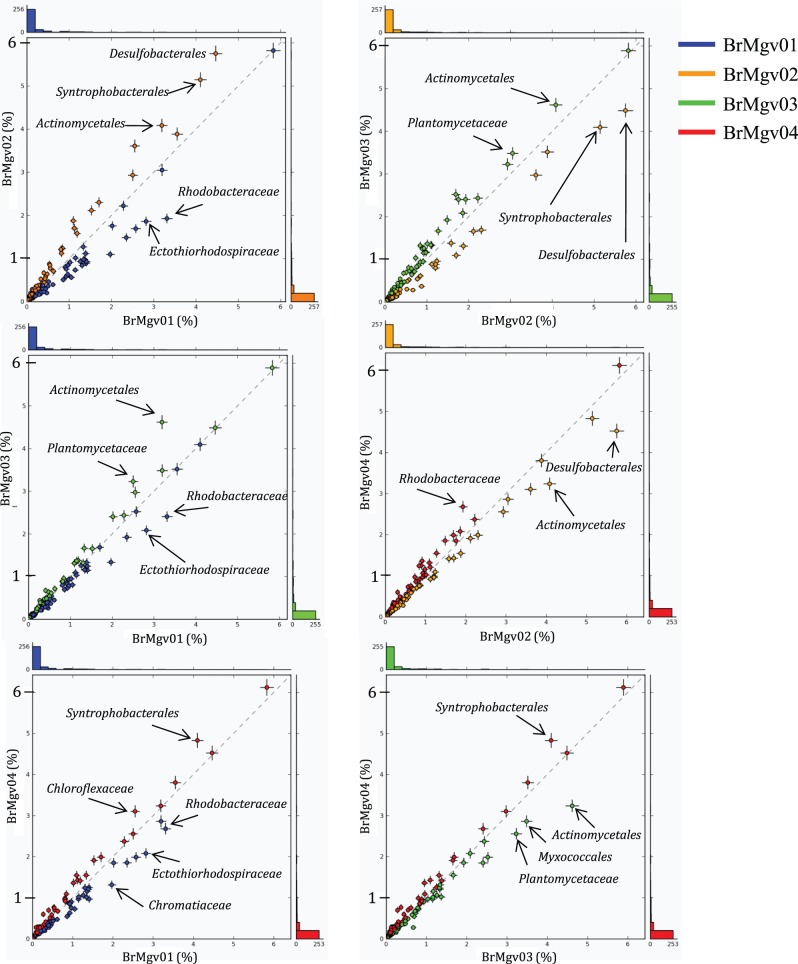
Profile scatter plot indicating the relative proportion of sequences at the 5 level (MG-RAST annotation) determined using STAMP software.

The phylogenetic affiliation of the sequences obtained in our study allowed a robust comparison of the taxonomically dominant groups in distinct mangroves (Figure 3). Pairwise comparisons of mangroves, as visualized by STAMP, showed a higher occurrence of *Planctomycetaceae* and *Actinomycetales* in BrMgv03. More hits affiliated with *Desulfobacterales* were observed in BrMgv02, where more *Syntrophobacterales*-related sequences also were found compared to samples from BrMgv01 and BrMgv03 (Figure 3). In BrMgv01, a higher incidence of *Rhodobacteraceae* was found when compared with BrMgv02 and BrMgv03. BrMgv04 had a higher incidence of sequences affiliated with *Syntrophobacterales* compared with BrMgv01 and BrMgv03 and of *Rhodobacteraceae* compared with BrMgv02.

What drives the observed variation cannot be fully explained by our data and experimental setting alone. However, considering the mangrove characteristics listed in Table 1, and based on the literature of mangrove sediments, some major differences among the four sampled mangroves might play a role in selection for different groups of organisms. For example, the activities of mangrove roots provide a source of oxygen and interfere directly with the redox potential of mangrove sediments [6]. Thus, BrMgv02 might experience lower oxygen availability than the other sites due to its less dense vegetation, as the oil spill reduced the number of trees and not all plant species are present in this area. This scenario could have led to selection for anaerobic bacteria such as *Desulfobacterales*. In the other sampled mangroves, particularly BrMgv03 (where the mangrove forest is very dense and diverse), the occurrence of microbial groups that need at least some oxygen (microaerophiles), such as *Actinomycetales* and *Planctomycetaceae*, could have increased.

### COG and KEGG Categories Found in Mangrove Sediment Datasets

**Figure 4:**
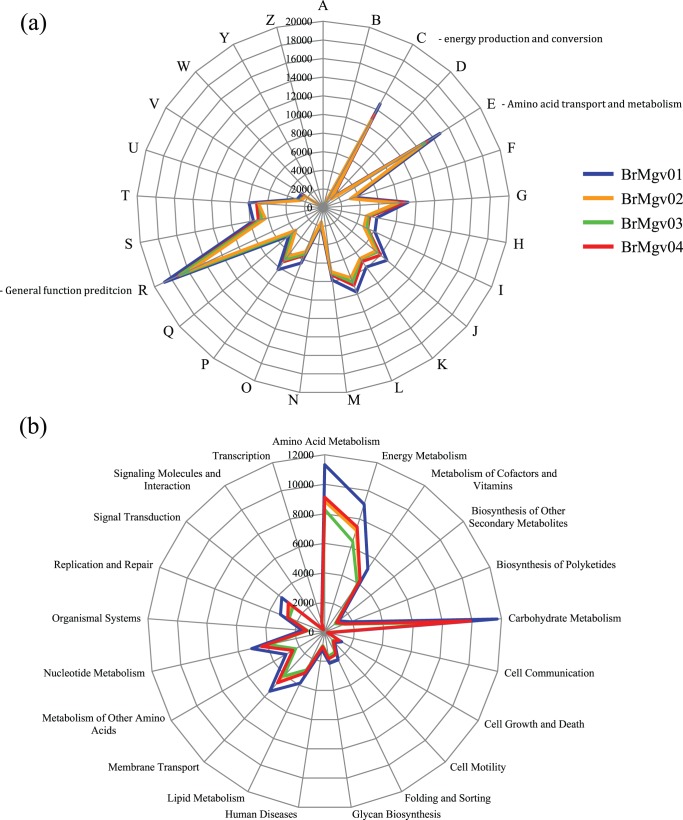
Functional assignment of metagenome sequences. (a) BLASTX analysis against the COGs database; read numbers were assigned to specific COG functional categories, and (b) BLASTX analysis against the NCBI-NR database conducted using MEGAN 4.0 software; reads numbers were assigned to specific KEGG identifiers.

The direct sequencing of environmental DNA has provided valuable insights into the lifestyle and metabolic capabilities of organisms inhabiting mangrove sediments. From the overall sequences in each metagenome, approximately 60% and 30% had matches in 25 COG and 23 KEGG categories, respectively (Figure 4).

At the level of COG categories, only slight variations between the metagenomes were observed. The dominant COGs confirmed the dominance of prokaryotic communities (i.e., Bacteria and Archaea) in mangrove sediments, with high abundances of sequences related to COG categories C, E, and R. Lower numbers of hits in other categories were observed for functions related to eukaryotic organisms (i.e., RNA processing, chromatin structure, etc.) (Figure 4a). Other researchers also have used the COG classification to attribute lifestyle characteristics to organisms [31]. The KEGG data indicated the presence of essential features for competitive microbial life within the mud in mangroves. For example, high occurrences of sequences related to amino acid metabolism, energy metabolism, metabolism of cofactors and vitamins, and carbohydrate metabolism were detected (Figure 4b).

### Metabolic Mapping of the Transformation of Carbon, Nitrogen, and Sulphur in Mangrove Sediments

**Figure 5:**
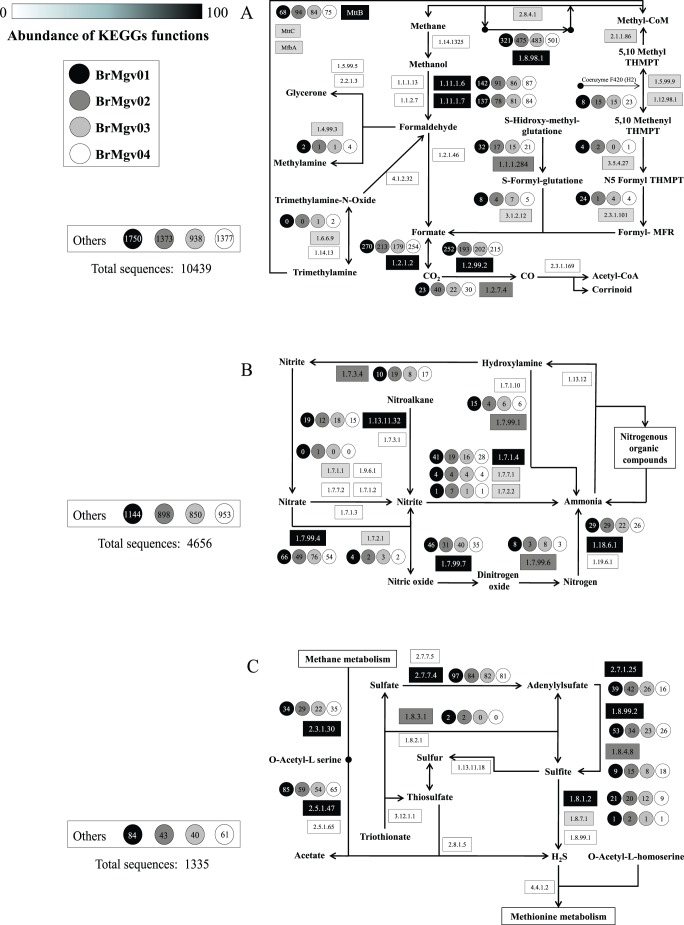
Part of a SEED-based functional analysis of mangrove metagenomes. Each item represents a functional role in the SEED and is labelled by the number of reads assigned in each dataset: (a) carbon fixation and methane metabolism; (b) nitrogen metabolism; and (c) sulphur metabolism. Boxes indicate the KEGG characteristic identified, and numbers in circles indicate the number of sequences from each metagenome affiliated with the KEGG function.

The affiliations of the sequences in the KEGG database allowed us to map the biogeochemical transformations that might possibly be performed by microbes in mangrove sediments (Figure 5). The variations in oxygen availability in mangrove sediments, which are promoted by the tidal regime, make this biome a special environment in which the transformations of compounds and nutrient cycling are adapted to the ever-shifting availability of the oxygen. Li et al. [15] studied the high variability of oxygen in mangroves and described the occurrence of ammonia-oxidizing Archaea and Bacteria in mangrove sediments. They reported that although mangrove sediments are predominantly anoxic, the constant shifting of aerobic and anaerobic conditions provides suitable environments for nitrification processes to occur, thereby possibly supplying nitrate for other nitrogen transformations such as denitrification and anaerobic ammonium oxidation (anammox). Dias et al. [20] also recently demonstrated the occurrence of ammonia-oxidizing Archaea in the Brazilian mangroves assessed in the present study.

#### Carbon metabolism (methane metabolism)

Although no hits were observed for genes involved in methanotrophy, the further transformation of methanol into formaldehyde and then formate was suggested by the metabolic reconstruction. The annotation of sequences indicated that genes required for aerobic and anaerobic respiratory activities of microbes in mangroves were present in mangroves (Figure 5a), possibly responding to the high generation of carbon dioxide mainly from the metabolism of trimethylamine (a precursor of trimethylamine oxide), which is converted into formaldehyde and later generates formate. A high occurrence of genes involved in the conversion of carbon dioxide into carbon monoxide and later into acetyl-CoA also was detected. The distinct mangrove datasets differed only in the processes involved in the transformation of methanol into formaldehyde: a higher number of matches was found in BrMgv01 compared to the other three mangroves (Figure 5a).

#### Nitrogen metabolism

The annotation of sequences relevant to nitrogen metabolism revealed the presence of genes involved with nitrogen immobilization and mineralisation in mangrove sediments as well as insights into the mineral transformations of nitrogen (Figure 5b). First, sequences related to atmospheric nitrogen fixation were present in the datasets, which corroborates data from the literature that describe the role of diazotrophs in mangrove sediments [54]. In contrast, sequences related to nitrification were not observed, although a high occurrence of sequences related to genes involved in the transformation of nitrate was found. In this case, the existence of distinct mechanisms for nitrate transformation could be observed, with sequences affiliated with genes related to the dissimilatory reduction of nitrate (DRNA) and also sequences of genes related to the transformation of nitrate into nitric oxide, dinitrogen oxide, and later into nitrogen (denitrification). The balance among these pathways is influenced greatly by environmental conditions, such as temperature, oxygen, nitrate availability, and organic matter content in the sediment [55]. Whether other genes, e.g. those related to anammox, are present but not detected due to the low density of such organisms remains an issue that needs to be better addressed. Overall, the numbers of sequences affiliated with each of the described functions were similar among the four analysed metagenomes.

#### Sulphur transformations

The sulphur transformation data indicate that the most predominant type of sulphur metabolism occurring in the sediments generates the reductive form of this compound (sulfite and H_2_S) (Figure 5c). Most of the genes observed were related to the conversion of sulphate into adenylylsulphate and to the further generation of sulfite and H_2_S. The reduction of sulfite into H_2_S seems to be an important transformation in mangroves, as all of the KEGG functions involved in this step were detected. However, the H_2_S generated is not further transformed in mangrove sediment (except for 4 sequences in the BrMgv02 dataset); the H_2_S might be released by volatilisation, thus producing the typical smell of mangroves [56]. Sulphate-reducing bacteria are important in organic matter degradation in anoxic environments. In marine sediments from temperate climates, these organisms perform 53% of organic matter degradation, and the values vary between 70% and 90% in salt marsh plateaus [4]. In mangrove sediments, the organisms related to sulphate reduction are *Deltaproteobacteria*; these organisms are abundant, possibly indicating the importance of such metabolism in the mangrove environment.

Overall, the metabolisms of carbon, nitrogen, and sulphur are coupled together within the microbial cells, and this is particularly true for the transformations of sulphur and carbon. The prevalent lack of oxygen coupled with the abundance of organic matter in mangrove sediments generates an optimal environment for the development of several anaerobic organisms, such as sulphate-reducing bacteria and methanogens [14], [57]. These groups share the same niche and follow a gradient according to substrate availability [58]. Simple substrates (e.g., methanol and mono-, di-, and trimethylamine) are important for methanogens [56], whereas sulphate-reducing bacteria are capable of degrading more complex substrates, such as long-chain and aromatic hydrocarbons [59].

### Major Hosts for Genes Involved in Biogeochemical Cycles in Mangrove Sediments

**Figure 6:**
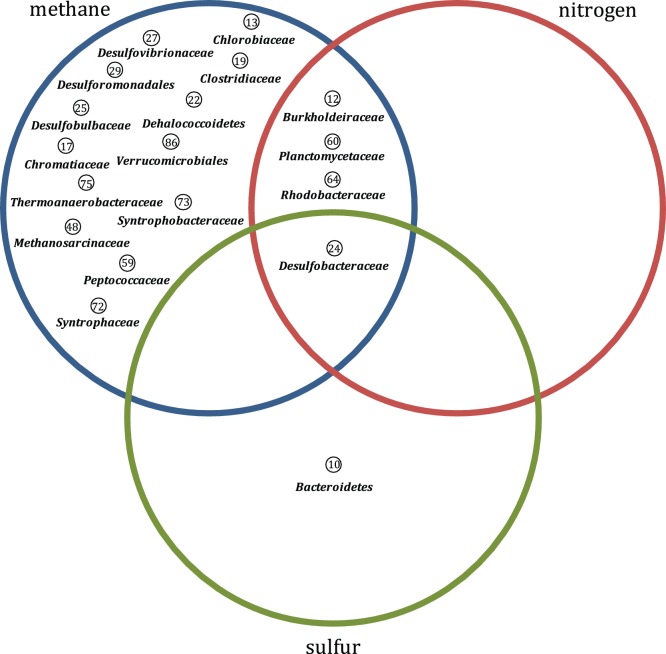
Taxonomic affiliation of main microbial groups involved in methane, nitrogen, and sulphur transformations in mangroves. Reads assigned by MEGAN 4.0 software were based on BLASTX vs. NCBI-NR. Numbers refer to the complete table which is part Supplementary Figure S2.

Although the four mangrove datasets varied only slightly in terms of the observed metabolic transformations, the taxonomic affiliations of sequences revealed the phylogeny of microbial groups harbouring the machinery involved in these biogeochemical cycles (Figure 6 and Supplementary Figure S2). Comparison of the distinct datasets revealed some differences in the identities of microbes possibly acting in distinct mangroves (Supplementary Figure S2).

In general, the majority of organisms identified in the evaluated transformations were Proteobacteria, Clostridia, and Firmicutes. However, Deltaproteobacteria (Desulfobacteraceae, Desulfobulbaceae, Desulfovibrionaceae, Geobacteraceae, Syntrophaceae, and Syntrophobacteraceae) were greatly represented in methane transformations; nitrogen transformations possibly were conducted by Planctomycetes, Alphaproteobacteria, Betaproteobacteria, and Deltaproteobacteria; and sulphur metabolism was mainly represented by groups of Bacteroidetes and Desulfobacteraceae.

In conclusion, the analyses of the taxonomic groups with genes involved in these biogeochemical cycles in all of the studied mangroves allowed us to tentatively describe the ‘microbial core for mangrove functioning’, which mainly was composed of *Desulfobacteraceae* (harbouring genes involved in all three of the analysed cycles), and other three groups involved in methane and nitrogen cycles (*Rhodobacteraceae*, *Planctomycetaceae*, and *Burkholderiacea*e) (Figure 6).

### Comparison of Mangrove Metagenomes with Other Metagenomes

**Figure 7:**
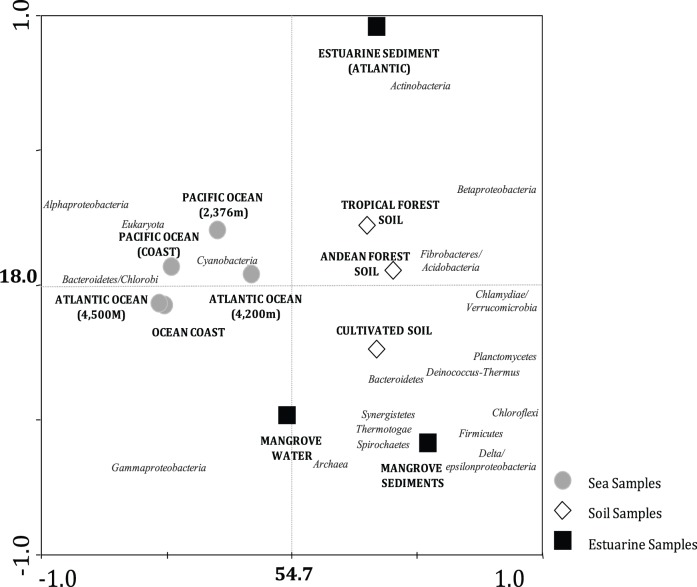
Principal component analysis of metagenomes based on taxonomic affiliation of reads determined using MG-RAST. The percentages of variance explained in each axis are indicated.

In order to characterize the microbial groups found in the mangrove metagenomes, we compared our dataset against a collection of selected metagenomes from other environments. In this analysis, the complete mangrove dataset was used as one sample representing the mangrove metagenome. The obtained plot achieved a high level of variance explanation, with values of 54.7% for the first axis (x) and 18.0% for the second axis (y) (Figure 7). This plot shows that the metagenomes from soils and oceans lie along the first axis, whereas the samples from mangroves lie at the middle of the axis, indicating the co-occurrence of groups found in these two groups (soils and ocean) in mangroves (Figure 7). The metagenome from mangrove sediments generated in this study was placed more on the side of the soil samples, but its fidelity to other soils from database was not completely observed in the second axis separation. In this case, it is observed the separation of mangrove sediments form other soils by the allocation of the mangrove sample far from the middle of the axis.

Besides the samples separation, it is also possible to determine the microbial taxa with differential occurrence in the compared environments (Supplementary Figure S3). Along the first axis, the separations were based on the more common occurrence of *Alphaproteobactera*, *Eukayota*, *Cyanobacteria*, and *Bacteroidetes/Chlorobi* in ocean samples and *Betaproteobacteria*, *Fibrobacteres/Acidobacteria*, *Planctomycetes*, *Deinococcus-Thermus*, and *Bacteroidetes* in soils. For the separation observed on the second axis, the microbial taxa involved were the *Actinobacteria* (mostly found in estuarine sediment), *Gammaproteobacteria* and *Archaea* (more commonly occurring in the mangrove water dataset), and *Synergistetes*, *Thermotogae*, *Spirochaetes*, and *Delta/Epsilonproteobacteria* in mangrove sediments (Figure 7). This result again links the high occurrence of *Deltaproteobacteria* with the niches available for microbial colonization in mangrove sediments, making these groups candidates for more thorough assessment in future studies of mangrove microbiology.

### Concluding Remarks and Future Perspectives

This work represents a first effort to better understand mangrove microbiology and potential metabolic pathways by metagenomics. The methodology applied in this survey provides a first look at the genetic basis that underlies the biogeochemical transformations that occur in this environment. We discovered that the besides the particular composition of the fauna and flora in the mangrove, their sediments also have a particular microbiome specific to this environment. Future work should focus on a complete description of the potential metabolic ways of these organisms, and important advances will be achieved by applying metatranscriptomics (i.e., biochemical-based studies) in mangrove sediments. The public availability of these metagenomes will serve as a basis for comparison with other distinct environments, which in turn will allow for a more complete view of microbiomes inhabiting distinct ecosystems.

## Supporting Information

Figure S1 Percentage of classified reads assigned to phylogenetic groups determined by comparison against distinct databases. (a) BLASTX analysis of data against the Pfam database using WebCARMA software (cut-off E-value 1e-10). (b) BLASTX analysis of data against the NCBI-NR database using MEGAN 4.0 software (cut-off E-value 1e-10).
(EPS)

Figure S2 Microbial groups involved in the transformations of (a) methane, (b) nitrogen, (c) and sulphur in each mangrove dataset. Reads were assigned by MEGAN 4.0 software based on BLASTX vs. NCBI-NR.
(EPS)

Figure S3 Phylogenetic affiliation of metagenomic reads determined by BLASTX analysis against the SEED database using MG-RAST software (cut-off E-value 1e-10). This figure shows the taxonomic comparison of land and sea metagenomes.
(TIFF)

Table S1 Number of SSU rDNA sequences affiliated with phylogenetic groups using distinct databases.
(DOCX)

Table S2 Number of sequences classified by functional assignment using SEED identifiers in MG-RAST and MEGAN 4.0.
(DOCX)

Table S3 Metagenomes used for comparisons and their characteristics (taxonomy assignment was performed using MG-RAST).
(DOCX)
