# Supplementary figures and images for: The Microbiome of Brazilian Mangrove Sediments as Revealed by Metagenomics
Source: PLoS One. 2012 Jun 21;7(6):e38600. doi: 10.1371/journal.pone.0038600 (PMC3380894; doi:10.1371/journal.pone.0038600)

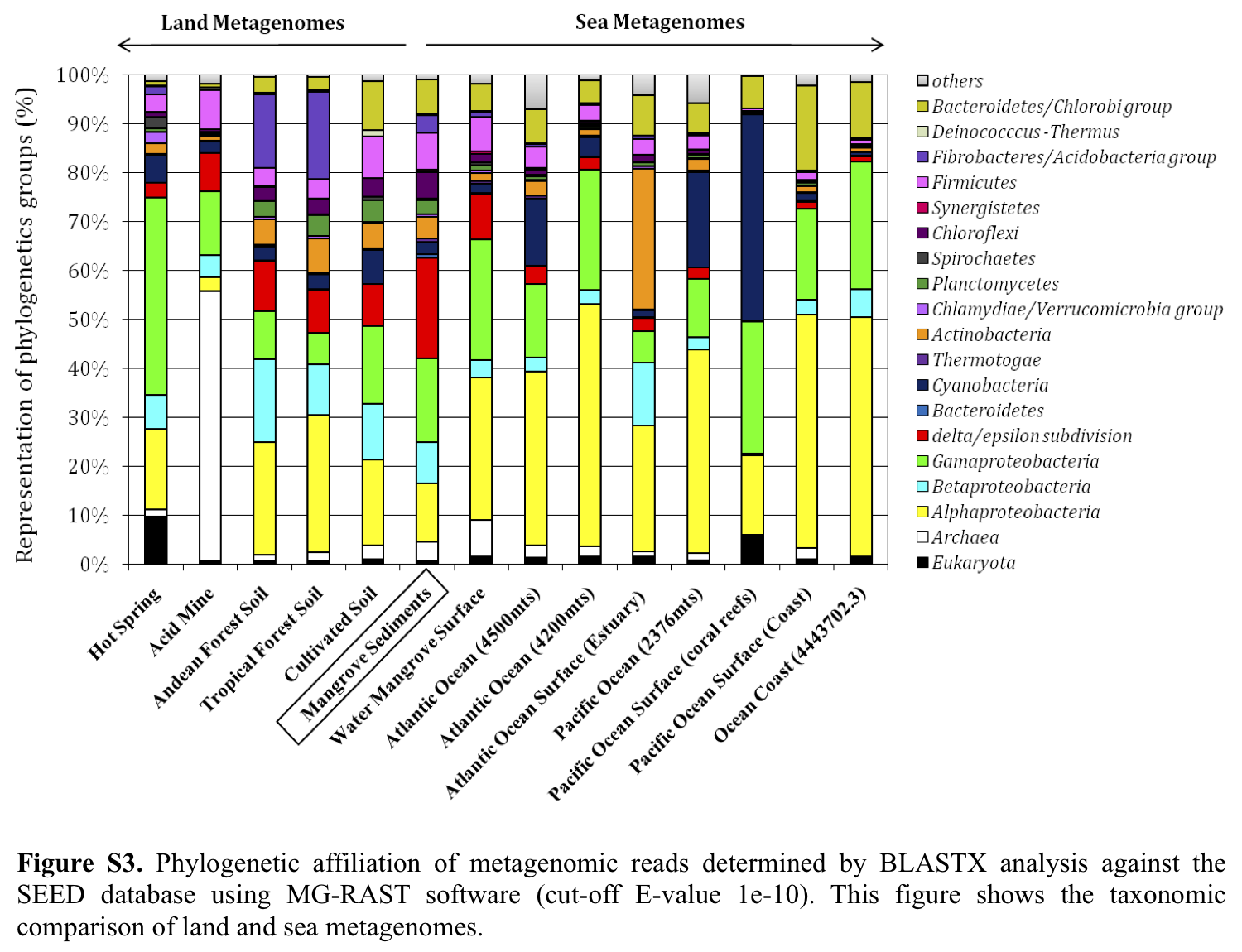

Supplement: Figure S3 — Phylogenetic affiliation of metagenomic reads determined by BLASTX analysis against the SEED database using MG-RAST software (cut-off E-value 1e-10). This figure shows the taxonomic comparison of land and sea metagenomes. (TIFF) [file pone.0038600.s003.tif]
