# Supplementary material for: The Microbiome of Brazilian Mangrove Sediments as Revealed by Metagenomics
Source: PLoS One. 2012 Jun 21;7(6):e38600. doi: 10.1371/journal.pone.0038600 (PMC3380894; doi:10.1371/journal.pone.0038600)
Supplement: Table S1 — Number of SSU rDNA sequences affiliated with phylogenetic groups using distinct databases. (DOCX) [file pone.0038600.s004.docx]

**Table S1.** Number of SSU rDNA sequences affiliated with phylogenetic groups using distinct databases

|  | **Databases** | | | | | | | | | | | | | |
| --- | --- | --- | --- | --- | --- | --- | --- | --- | --- | --- | --- | --- | --- | --- |
|  | **RDP** | | | |  | **Greengenes** | | | |  | **GenBank** | | | |
|  | BrMgv01 | BrMgv02 | BrMgv03 | BrMgv04 |  | BrMgv01 | BrMgv02 | BrMgv03 | BrMgv04 |  | BrMgv01 | BrMgv02 | BrMgv03 | BrMgv04 |
| *Proteobacteria* | 61 | 43 | 45 | 40 |  | 61 | 43 | 44 | 40 |  | 58 | 41 | 44 | 39 |
| *Firmicutes* | 12 | 9 | 11 | 9 |  | 13 | 9 | 12 | 9 |  | 12 | 8 | 12 | 10 |
| *Actinobacteria* | 6 | 10 | 7 | 7 |  | 6 | 9 | 7 | 7 |  | 6 | 8 | 8 | 7 |
| *Bacteroidetes* | 11 | 4 | 3 | 10 |  | 10 | 4 | 3 | 10 |  | 11 | 4 | 3 | 9 |
| *Chloroflexi* | 6 | 4 | 1 | 4 |  | 6 | 5 | 1 | 4 |  | 6 | 3 | 0 | 4 |
| *Planctomycetes* | 2 | 1 | 3 | 2 |  | 2 | 0 | 4 | 2 |  | 2 | 1 | 4 | 4 |
| *Cyanobacteria* | 2 | 1 | 2 | 3 |  | 3 | 2 | 1 | 3 |  | 3 | 1 | 1 | 1 |
| *Euryarchaeota* | 0 | 3 | 0 | 3 |  | 3 | 3 | 2 | 4 |  | 3 | 3 | 2 | 4 |
| *Acidobacteria* | 3 | 0 | 1 | 2 |  | 3 | 0 | 1 | 2 |  | 3 | 0 | 1 | 1 |
| *Spirochetes* | 2 | 3 | 0 | 0 |  | 2 | 2 | 0 | 0 |  | 2 | 3 | 0 | 0 |
| *Nitrospira* | 0 | 1 | 2 | 1 |  | 0 | 1 | 2 | 1 |  | 0 | 1 | 2 | 1 |
| *Verrucomicrobia* | 1 | 0 | 1 | 2 |  | 1 | 1 | 2 | 2 |  | 1 | 1 | 2 | 2 |
| *Synergistetes* | 1 | 1 | 0 | 0 |  | 0 | 1 | 0 | 0 |  | 0 | 1 | 0 | 0 |
| *Tenericutes* | 0 | 1 | 0 | 1 |  | 1 | 1 | 0 | 1 |  | 0 | 1 | 0 | 1 |
| *Crenarchaeota* | 1 | 0 | 0 | 0 |  | 0 | 0 | 0 | 0 |  | 0 | 0 | 0 | 0 |
| *Deinococcus-Thermus* | 0 | 0 | 1 | 0 |  | 0 | 0 | 1 | 0 |  | 0 | 0 | 1 | 0 |
| *Gemmatimonadetes* | 0 | 1 | 0 | 0 |  | 0 | 1 | 0 | 0 |  | 0 | 1 | 0 | 0 |
| *Aquaficae* | 0 | 0 | 0 | 1 |  | 0 | 0 | 0 | 0 |  | 0 | 0 | 0 | 0 |
| *Eukaryota* | 0 | 0 | 0 | 0 |  | 0 | 0 | 0 | 0 |  | 0 | 0 | 0 | 2 |
| Unclassified | 3 | 0 | 3 | 0 |  | 0 | 0 | 0 | 0 |  | 4 | 5 | 0 | 0 |
| Total | 111 | 82 | 80 | 85 |  | 111 | 82 | 80 | 85 |  | 111 | 82 | 80 | 85 |
|  |  |  |  |  |  |  |  |  |  |  |  |  |  |  |
| *Alphaproteobacteria* | 11 | 8 | 5 | 3 |  | 11 | 7 | 5 | 3 |  | 10 | 9 | 5 | 3 |
| *Betaproteobacteria* | 4 | 4 | 1 | 3 |  | 4 | 4 | 1 | 3 |  | 4 | 4 | 1 | 3 |
| *Deltaproteobacteria* | 18 | 16 | 14 | 16 |  | 18 | 16 | 13 | 16 |  | 18 | 15 | 13 | 16 |
| *Epsilonproteobacteria* | 2 | 1 | 9 | 3 |  | 2 | 1 | 9 | 3 |  | 2 | 0 | 8 | 3 |
| *Gammaproteobacteria* | 26 | 14 | 16 | 15 |  | 26 | 14 | 16 | 15 |  | 24 | 13 | 16 | 14 |
| Unclassified | 0 | 0 | 0 | 0 |  | 0 | 1 | 0 | 0 |  | 0 | 0 | 1 | 0 |
|  | 61 | 43 | 45 | 40 |  | 61 | 43 | 44 | 40 |  | 58 | 41 | 44 | 39 |
