# Supplementary material for: The Microbiome of Brazilian Mangrove Sediments as Revealed by Metagenomics
Source: PLoS One. 2012 Jun 21;7(6):e38600. doi: 10.1371/journal.pone.0038600 (PMC3380894; doi:10.1371/journal.pone.0038600)
Supplement: Table S2 — Number of sequences classified by functional assignment using SEED identifiers in MG-RAST and MEGAN 4.0. (DOCX) [file pone.0038600.s005.docx]

**Table S2.** Number of sequences classified by functional assignment using SEED identifiers in MG-RAST and MEGAN 4.0.

|  | **MEGAN 4.0** | | | | **MG-RAST** | | | |
| --- | --- | --- | --- | --- | --- | --- | --- | --- |
| **SEDD Identifier** | BrMgv01 | BrMgv02 | BrMgv03 | BrMgv04 | BrMgv01 | BrMgv02 | BrMgv03 | BrMgv04 |
| Carbohydates | 8,403 | 7,154 | 6,290 | 7,336 | 7,319 | 6,544 | 6,379 | 5,407 |
| Clustering-based subsystems | 2,322 | 1,965 | 1,835 | 2,010 | 7,376 | 6,001 | 6,511 | 5,282 |
| Amino Acids and Derivatives | 5,185 | 4,048 | 3,883 | 4,157 | 4,862 | 4,020 | 3,972 | 3,431 |
| Protein Metabolism | 5,376 | 4,352 | 3,785 | 4,426 | 4,543 | 3,907 | 3,590 | 3,254 |
| Respiration | 5,198 | 4,169 | 3,720 | 4,358 | 3,866 | 3,381 | 2,972 | 2,836 |
| Virulence | 6,672 | 5,161 | 4,949 | 5,307 | 3,150 | 2,426 | 2,476 | 2,040 |
| Cofactors, Vitamins, Prosthetic Groups, Pigments | 3,415 | 2,858 | 2,441 | 2,904 | 2,688 | 2,352 | 2,203 | 1,908 |
| DNA Metabolism | 3,798 | 3,310 | 3,049 | 3,229 | 2,169 | 2,003 | 1,792 | 1,487 |
| Cell Wall and Capsule | 3,415 | 2,622 | 2,409 | 2,839 | 2,069 | 1,723 | 1,693 | 1,534 |
| RNA Metabolism | 2,786 | 2,126 | 1,989 | 2,317 | 1,927 | 1,601 | 1,520 | 1,374 |
| Nucleosides and Nucleotides | 1,839 | 1,467 | 1,431 | 1,599 | 1,680 | 1,400 | 1,442 | 1,236 |
| Membrane Transport | 1,248 | 1,136 | 879 | 1,067 | 1,364 | 1,229 | 1,199 | 997 |
| Stress Response | 2,363 | 1,827 | 1,618 | 1,806 | 1,352 | 1,107 | 1,149 | 980 |
| Regulation and Cell signaling | 1,481 | 1,049 | 1,108 | 1,177 | 1,246 | 886 | 1,025 | 808 |
| Motility and Chemotaxis | 1,470 | 1,303 | 1,149 | 1,241 | 1,047 | 990 | 895 | 800 |
| Phosphorus Metabolism | 873 | 742 | 611 | 731 | 899 | 791 | 701 | 646 |
| Cell Division and Cell Cycle | 1,536 | 1,342 | 1,104 | 1,356 | 866 | 739 | 698 | 630 |
| Sulfur Metabolism | 796 | 660 | 601 | 680 | 863 | 718 | 710 | 585 |
| Fatty Acids, Lipids, and Isoprenoids | 1,799 | 1,371 | 1,379 | 1,444 | 668 | 491 | 580 | 404 |
| Metabolism of Aromatic Compounds | 1,115 | 850 | 871 | 887 | 621 | 467 | 588 | 445 |
| Potassium metabolism | 174 | 139 | 110 | 122 | 471 | 319 | 360 | 346 |
| Nitrogen Metabolism | 1,278 | 972 | 1,037 | 1,007 | 437 | 317 | 432 | 245 |
| Miscellaneous | 655 | 481 | 476 | 543 | 242 | 163 | 208 | 196 |
| Macromolecular Synthesis | 0 | 0 | 0 | 0 | 67 | 34 | 41 | 31 |
| Secondary Metabolism | 307 | 280 | 257 | 280 | 55 | 29 | 36 | 34 |
| Photosynthesis | 10 | 27 | 21 | 18 | 4 | 18 | 8 | 9 |
| Phages, Prophages, Transposable elements | 103 | 95 | 90 | 92 | 2 | 0 | 5 | 0 |
| Dormancy and Sporulation | 32 | 37 | 26 | 34 | 0 | 0 | 0 | 0 |
| Unclassified sequences | 186,284 | 179,690 | 167,803 | 164,638 | 198,080 | 187,577 | 171,736 | 180,660 |
