# Supplementary material for: The Microbiome of Brazilian Mangrove Sediments as Revealed by Metagenomics
Source: PLoS One. 2012 Jun 21;7(6):e38600. doi: 10.1371/journal.pone.0038600 (PMC3380894; doi:10.1371/journal.pone.0038600)
Supplement: Table S3 — Metagenomes used for comparisons and their characteristics (taxonomy assignment was performed using MG-RAST). (DOCX) [file pone.0038600.s006.docx]

**Table S3.** Metagenomes used for comparisons and their characteristics (taxonomy assignment was performed using MG-RAST)

| **Ecosystem** | **MG-RAST code** | **Number of reads** | **Sequencing methodology** | **Classified reads** | **Reference** |
| --- | --- | --- | --- | --- | --- |
| Mangrove Sediments | 4452857.3 | 905,521 | 454 -pyrosequencing | 31.5 % | This study |
| Mangrove Water | 4441598.3 | 148,018 | Sanger - capillary | 75.7 % | Rusch et al. 2007 |
| Atlantic Ocean (surface) | 4441584.3 | 126,162 | Sanger - capillary | 80.1 % | Rusch et al. 2007 |
| Atlantic Ocean (4,200m) | 4441572.3 | 317,180 | Sanger - capillary | 82.4 % | Rusch et al. 2007 |
| Atlantic Ocean (4,500m) | 4441587.3 | 257,581 | Sanger - capillary | 82.5 % | Rusch et al. 2007 |
| Pacific Ocean (2,376m) | 4441594.3 | 102,708 | Sanger - capillary | 87.2 % | Rusch et al. 2007 |
| Pacific Ocean (coast) | 4443713.3 | 217,549 | 454 -pyrosequencing | 42.0 % | Unpublished |
| Pacific Ocean (coral reefs) | 4440039.3 | 289,723 | 454 -pyrosequencing | 1.3 % | Dinsdale et al. 2008 |
| Ocean Coast | 4443702.3 | 204,693 | 454 -pyrosequencing | 39.5 % | Unpublished |
| Tropical Forest Soil | 4446153.3 | 780,588 | 454 -pyrosequencing | 50.8 % | De Angelis et al. 2010 |
| Agricultural Soil | 4441091.3 | 138,347 | Sanger - capillary | 66.2 % | Tringe et al. 2005 |
| Andean Forest Soil | 4445417.3 | 618,540 | 454 -pyrosequencing | 43.7 % | Unpublished (Gebix group) |
